# Icaritin Alleviates Glutamate-Induced Neuronal Damage by Inactivating GluN2B-Containing NMDARs Through the ERK/DAPK1 Pathway

**DOI:** 10.3389/fnins.2021.525615

**Published:** 2021-02-22

**Authors:** Song Liu, Chaoming Liu, Lijiao Xiong, Jiali Xie, Cheng Huang, Rongbiao Pi, Zhihua Huang, Liangdong Li

**Affiliations:** ^1^Key Laboratory of Prevention and Treatment of Cardiovascular and Cerebrovascular Diseases of Ministry of Education, Gannan Medical University, Ganzhou, China; ^2^First Affiliated Hospital of Gannan Medical University, Ganzhou, China; ^3^Institute for Medical Sciences of Pain, Department of Physiology, School of Basic Medical Sciences, Gannan Medical University, Ganzhou, China; ^4^Department of Pharmacology and Toxicology, School of Pharmaceutical Sciences, Sun Yat-sen University, Guangzhou, China

**Keywords:** icaritin, NMDA receptor, GluN2B, GluN2A, ERK1/2/DAPK1 pathway

## Abstract

Excitatory toxicity due to excessive glutamate release is considered the core pathophysiological mechanism of cerebral ischemia. It is primarily mediated by N-methyl-D-aspartate receptors (NMDARs) on neuronal membranes. Our previous studies have found that icaritin (ICT) exhibits neuroprotective effects against cerebral ischemia in rats, but the underlying mechanism is unclear. This study aims to investigate the protective effect of ICT on glutamate-induced neuronal injury and uncover its possible molecular mechanism. An excitatory toxicity injury model was created using rat primary cortical neurons treated with glutamate and glycine. The results showed that ICT has neuroprotective effects on glutamate-treated primary cortical neurons by increasing cell viability while reducing the rate of lactate dehydrogenase (LDH) release and reducing apoptosis. Remarkably, ICT rescued the changes in the ERK/DAPK1 signaling pathway after glutamate treatment by increasing the expression levels of p-ERK, p-DAPK1 and t-DAPK1. In addition, ICT also regulates NMDAR function during glutamate-induced injury by decreasing the expression level of the GluN2B subunit and enhancing the expression level of the GluN2A subunit. As cotreatment with the ERK-specific inhibitor U0126 and ICT abolishes the beneficial effects of ITC on the ERK/DAPK1 pathway, NMDAR subtypes and neuronal cell survival, ERK is recognized as a crucial mediator in the protective mechanism of ICT. In conclusion, our findings demonstrate that ICT has a neuroprotective effect on neuronal damage induced by glutamate, and its mechanism may be related to inactivating GluN2B-containing NMDAR through the ERK/DAPK1 pathway. This study provides a new clue for the prevention and treatment of clinical ischemic cerebrovascular diseases.

## Introduction

Stroke is currently a prevalent disease that severely threatens human health and life expectancy. Cerebral ischemic stroke accounts for approximately 85% of all stroke cases (Mukherjee and Patil, 2011; Das et al., 2016; Rama and García, 2016). Recently, the incidence of cerebral apoplexy has increased annually and is on rise in younger adults (Meng et al., 2019). However, drugs for the treatment of ischemic stroke and improving the prognosis that have remarkable curative effects and few side effects remain scarce. Cerebral ischemia/reperfusion injury is a complex pathophysiological process that involves multiple events, such as brain energy disorder, cell acidosis, excitatory amino acid toxicity, intracellular calcium overload, free radical damage, inflammatory cytokine damage, and activation of apoptosis genes (Khoshnam et al., 2017; Meng et al., 2019; Wei et al., 2019). Among them, excitatory toxicity plays an important role in cerebral ischemia/reperfusion injury, especially in the early stage (Amantea and Bagetta, 2017; Huang et al., 2017b). Therefore, effective inhibition of excitatory toxicity is a key strategy for reducing neuronal damage in ischemic brain injury.

Excessive glutamate release is the principal mechanism that leads to excitatory toxicity injury in ischemic neurons. Upon binding of glutamate, the N-methyl-D-aspartate (NMDA) receptor becomes highly permeable to Ca^2+^, indicating its crucial role in excitatory toxicity injury. Despite decades of clinical research, the use of NMDA receptor antagonists to treat cerebral ischemia have revealed uncertain efficacy or serious adverse reactions, since NMDARs are required for fundamental processes in the central nervous system (Mehta et al., 2007; Yu et al., 2015; Sun et al., 2018a). Recently, researchers proposed that NMDA receptors can be divided into synaptic and extrasynaptic NMDARs according to their locations on the cell membrane of the postsynaptic neuron (Hardingham and Bading, 2010). The two subtypes of receptors are differentiated by their subunits: GluN2A-containing NMDA receptors that are mainly distributed at the synapse contribute to normal physiological functions and promote survival of neurons; GluN2B-containing NMDA receptors that are primarily found in extrasynaptic locations, mediate excitatory toxic injury caused by various noxious stimulations including cerebral ischemia, and promote neuronal death (Liu et al., 2007; Sun et al., 2015b). Therefore, targeting the specific subtype of NMDAR could contribute to the inhibition of excitatory toxicity during cerebral ischemia while maintaining the normal function of NMDAR.

Icaritin (ICT) is a phytoestrogen that can be extracted from the dry stems and leaves of plants in the genus Epimedium, including *Epimedium sagittatum*, *Epimedium pubescens*, *Epimedium wushanense*, and *Epimedium koreanum*. Studies have found that ICT has anti-tumor (Sun et al., 2015a), anti-osteoporosis (Jiang et al., 2014), anti-liver fibrosis (Liu et al., 2014) effects, among others. Icariin, the precursor of ICT, protects oxygen-glucose deprivation-injured cortical neurons by increasing deacetylase levels (Zhu et al., 2010), improves cognitive impairment caused by chronic cerebral ischemia (Xu et al., 2009), and mitigates cognitive damage caused by transient cerebral ischemia/reperfusion (Zheng et al., 2008). All this evidence suggests that ICT may be a potential neuroprotective agent. Therefore, we conducted a preliminary study on the role of ICT in stroke and found that ICT is able to alleviate cerebral injury induced by middle cerebral artery occlusion (MCAO) reperfusion in rats. However, the mechanisms of action are still unclear.

Many studies have shown that the GluN2B subunit, as an important regulatory subunit of the NMDA receptor complex, mediates ischemic neuronal injury when phosphorylated at S1303 (Khoshnam et al., 2017; Shi et al., 2017; Tang et al., 2018; Zhao et al., 2018). This phosphorylation has been found to be inhibited by the extracellular signaling regulatory kinases/death associated protein kinase 1 (ERK/DAPK1) pathway (Xiong et al., 2018). In addition, icariin, the precursor of ICT, has the ability to promote the proliferation, differentiation, and migration of bone mesenchymal stem cells and osteoblasts by activating the ERK signaling pathway (Song et al., 2013; Qin et al., 2015; Wu et al., 2015). Therefore, we speculate that ICT may activate the ERK/DAPK1 pathway, inhibit the phosphorylation of GluN2B, and reduce the function of extrasynaptic NMDA receptors. This study intends to discover the protective effects of ICT on glutamate-induced neuronal injury in primary cultured cortical neurons and whether the ERK/DAPK1 signaling pathway is involved in the regulation of GluN2B-containing NMDA receptors.

## Materials and Methods

### Animals and Drugs

The experiments were performed in accordance with the standard biosecurity and institutional safety procedures of Gannan Medical University (Ganzhou China). Male and female Sprague-Dawley rats weighing 250-280 g were purchased from Hunan Slack Jingda Experimental Animal Co., Ltd. (Animal Center License No. SCXK (Xiang) 2016-0002, Animal Qualification No. 43004700035146, SPF level); they were housed in a temperature-controlled (22-24°C) room with 60-70% humidity in 12-hour light-dark cycles. The rats had free access to drinking water and a standard rodent diet. All animal experiments were performed under the guidelines of the Animal Care and Use Committee of Gannan Medical University (Ganzhou China). Icaritin (ICT) (C_21_H_20_O_6_, MW = 368.38 g/mol, purity ≥ 99%), a yellow crystalline powder, was supplied by Shanghai Tianshui Chemical Co., Ltd., and it was dissolved in dimethyl sulfoxide (DMSO, D2650, Sigma, United States). U0126 (S1102, Selleckchem) was dissolved in DMSO to produce a 500 μM stock solution.

### Primary Cortical Neuron Culture

Primary rat cerebral cortical neurons were cultured as previously described (Li et al., 2017a). Briefly, pregnant SD rats at embryonic day 18 were euthanized by cervical dislocation after receiving 5% isoflurane anesthesia. Then, the skin over the abdomen was disinfected with 75% ethanol. Embryos were removed aseptically from the uterus, and the brain tissues were dissected. After removing blood vessels and meninges, the cortex was isolated and washed 3 times with Hank’s balanced salt solution (HBSS) before being cut into 1 mm cubes. These tissue cubes were then digested with 0.25% trypsin (Gibco) for 30 min at 37°C. Digestion was stopped with DMEM (Gibco) containing 10% fetal bovine serum (FBS, Gibco). After carefully pipetting the brain tissue, it was filtered with a 40 μm cell strainer (Fisherbrand), and a single cell suspension was obtained. Cells were plated onto poly-l-lysine-coated plates or dishes and incubated at 37°C for 4 hours. The medium was then replaced with serum-free neurobasal medium supplemented with 2% B27 and 1% Glutamax (Gibco), which was followed by re-incubation for 8 days; half of the medium was changed every 3 days. One micromolar cytarabine was added to the medium on day 3 to inhibit the growth of non-neuronal cells. Cells were maintained at 37°C in a humidified atmosphere of 5% CO_2_.

### Glutamate-Induced Neuronal Injury Model and Drug Treatments

**FIGURE 1:**
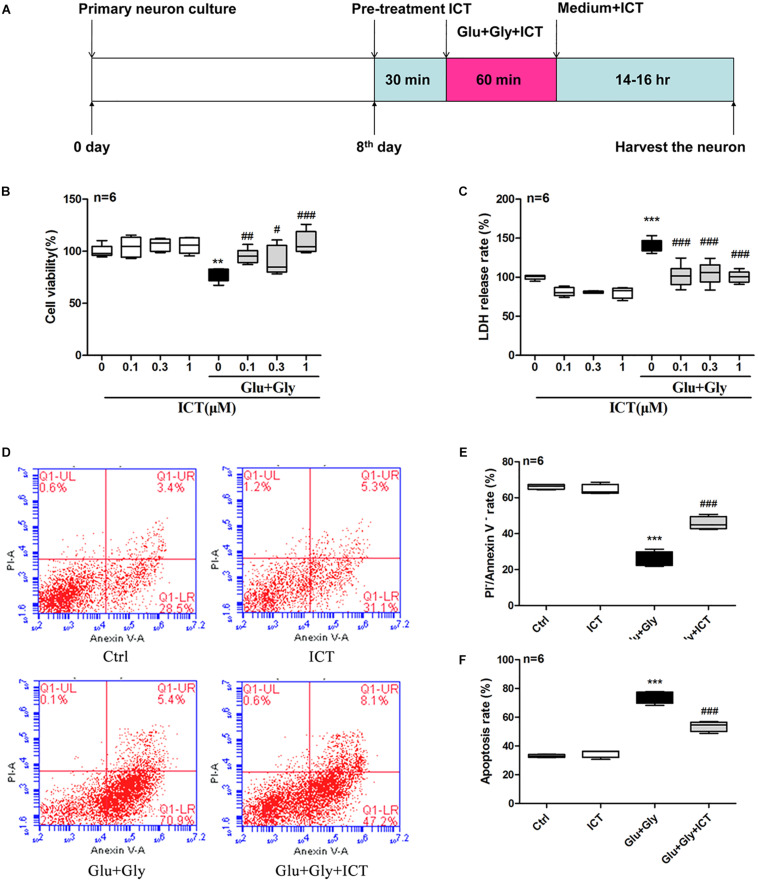
ICT increases cell viability and reduces the rate of LDH release and apoptosis in cortical neurons after glutamate-induced injury. Treatment timeline for the glutamate-induced injury model and for ICT treatment of rat primary cortical neurons **(A)**: ICT treatment started 30 min prior to the onset of glutamate injury and continued until the time neurons were harvested, which was 14-16 hours after the glutamate injury treatment. Cell viability **(B)** and LDH release rate **(C)** from the eight different treatment groups: control, 0.1 μM ICT, 0.3 μM ICT, 1 μM ICT, Glu+Gly, Glu+Gly+0.1 μM ICT, Glu+Gly+0.3 μM ICT, and Glu+Gly+1 μM ICT, were expressed as the mean ± SEM (*n* = 6). The annexin V/PI double staining results collected by flow cytometry are shown in scatter plots **(D)**. The percentages of surviving **(E)** and apoptotic **(F)** cells from the four treatment groups (control, 1 μM ICT, Glu+Gly and Glu+Gly+1 μM ICT) are shown as the mean ± SEM (*n* = 6). One-way ANOVA followed by Newman-Keuls test, **(B)**: F = 8.832, **(C)**: F = 30.18, **(E)**: F = 142.5, **(F)**: F = 151.2; **p < 0.01 and ***p < 0.001 vs. control; ^#^*p* < 0.05, ^##^*p* < 0.01 and ^###^*p* < 0.001 vs. Glu+Gly.

The primary cortical neurons were cultured for 8 days in cell culture medium containing ICT of different concentrations for 30 min before being washed once with Earle’s balanced salt solution (EBSS) solution. The medium was then replaced with EBSS containing 100 μM glutamate, 10 μM glycine, and ICT of different concentrations in the incubator with 5% CO_2_ at 37°C. After 1 h, the EBSS was replaced with new culture medium without phenol red (for the cell toxicity test) or with the original medium. These cells were cultured in the incubator for 14–16 h before further tests (Figure 1). For experiments with U0126 treatment, 5 μM U0126 (Cheemala et al., 2019) was added to the cell culture medium at the same time as ICT (Figure 4).

### Cell Viability Assay

Neurons cultured in 96-well plates at a concentration of 1 × 10^5^ cells/well were treated with ICT (0.1, 0.3, and 1 μM) and glutamate as previously described. Fourteen hours after the treatment, 10 μL of Cell Counting Kit-8 (CCK-8) solution (Solarbio) was added to each well according to the manufacturer’s instructions. After incubation at 37°C with 5% CO_2_ for 3 h, the absorbance value of each well was measured at a 490 nm wavelength by a Multi-Mode Microplate Reader (Biotek Instruments, INC Winooski, Vermont, United States). The absorbance value of each well was calibrated by subtracting the value of an empty well.

### Cell Toxicity Assay

Neurons cultured in 24-well plates at a concentration of 3 × 10^5^ cells/well were treated with ICT (0.1, 0.3, and 1 μM) and glutamate as previously described. The levels of lactate dehydrogenase (LDH) in the cell culture medium and in the lysed cells were analyzed using a lactate dehydrogenase (LDH) kit (Promega) according to the manufacturer’s instructions. The LDH release rate, which is an indicator of cell toxicity, was calculated using the following formula: 100% × LDH content in the supernatant/(LDH in supernatant + LDH in lysed cells).

### Apoptosis Analysis

Neurons cultured in 35 mm dishes at a concentration of 1.5 × 10^6^ cells/dish were treated with 1 μM ICT and glutamate. After 14 hours, the number of apoptotic cells was measured by flow cytometry using a FITC annexin V Apoptosis Detection kit (BD Pharmingen) according to the manufacturer’s instructions. The percentages of living cells (annexin V^–^/PI^–^) and apoptotic cells (annexin V^+^/PI^+^ and annexin V^+^/PI^–^) were calculated by CellQuest software (BD Biosciences).

### Immunofluorescent Staining

Fourteen hours after 1 μM ICT treatment was performed (as previously described), the cells cultured on cover glass slides in 24-well plates (1 × 10^5^/well) were fixed with 4% paraformaldehyde, washed in 0.1 M PBS for 20 min and permeabilized by 0.3% Triton X-100 for 10–15 min. They were then washed 3 times using PBS for 5 min and then nonspecific antigens were blocked using 3% bovine serum albumin (BSA) for 30 min at room temperature. The cells were subsequently immunostained with primary antibodies against NeuN (ab177487, Abcam), GluN2A (ab227233, Abcam) and GluN2B (ab65783, Abcam). After overnight incubation at 4°C, the samples were incubated for 90 min with the following secondary antibodies: Alexa-594 or Alexa-488 IgG (Alexa-594 for red and Alexa-488 for green, 1:2000; Invitrogen, Carlsbad, CA, United States). Negative controls were prepared with identical conditions, except without primary antibodies. Images were acquired using a laser scanning confocal microscope (Carl Zeiss Microscopy GmbH).

### Quantitative RT-PCR

The mRNA levels of GluN2B, GluN2A and DAPK1 were detected by quantitative real-time polymerase chain reaction (RT-qPCR). Total RNA was isolated from neurons cultured in 60 mm dishes (4.5 × 10^6^/well) using a TRIzol RNA Mini kit (Ambion) according to the manufacturer’s instructions. RNA concentration and purity were measured using a Multi-Mode Microplate Reader (Biotek Instruments, INC Winooski, Vermont, United States). The A260/280 and A260/230 ratios were used to assess the quality and quantity of total RNA. Four micrograms of RNA was used for cDNA synthesis using a Reverse Transcription Master Mix kit (Invitrogen). RT-qPCR was performed by using EvaGreen on the BioMark HD Nanofluidic qPCR System combined with GE 96.96 Dynamic Array IFC System. PCR was performed in reaction volumes of 20 μL, containing the following: 2 μL of cDNA, 10 μL of 1x SYBR^®^, Select Master Mix (Life Technologies), 2 μL of 10 μM forward and reverse primers, and 6 μL of ultra-pure water. Denaturation was carried out at 95°C for 10 s, annealing at 61°C for 25 s, and elongation at 72°C for 25 s for a total of 40 cycles. All samples were run in triplicate. Data analysis was performed following the manufacturer’s instructions (Fluidigm Real-Time PCR Analysis Software version 3.1.3). Relative expression levels of the genes were calculated using the 2^–Δ^ ^Δ^ ^*CT*^ method, comparing gene expression to that of β-actin, an endogenous control gene.

### Western Blot

Protein samples were collected from neurons cultured in 60 mm dishes (4.5 × 10^6^/well) that were treated with lysis buffer (50 mM Tris–HCl pH 7.5, 250 mM NaCl, 10 mM EDTA, 0.5%, NP-40, 10 μM leupeptin, 1 mM PMSF, and 4 mM NaF). Thirty micrograms of total protein was separated by 10% SDS-PAGE and then was transferred to PVDF membranes (Millipore, CA, United States). The membranes were blocked with 5% skim milk and then were incubated with primary antibodies against GluN2A (1:400, ab227233, Abcam, United Kingdom), p-GluN2B (1:200, ab81271, Abcam, United Kingdom), GluN2B (1:400, ab65783, Abcam, United Kingdom), p-DAPK1 (1:200, D4941, Sigma, United States), DAPK1 (1:400, 3008S, Cell Signaling Technology, United States), p-ERK (1:200, 4370S, Cell Signaling Technology, United States), ERK (1:200, sc93, Santa Cruz Biotechnology, United States) and β-tubulin (1:1000, K106281P, Solarbio, China). After washing with TBS-T for 5 min for a total of 3 times, HRP-conjugated secondary antibodies (goat anti-rabbit IgG, rabbit anti-goat IgG, and goat anti-mouse IgG) were applied to the blots. The blots were developed using ECL Western blotting detection reagents (Thermo Fisher Scientific, Waltham, MA, United States). The band densitometry was analyzed using an Amersham Imager 600 system.

### Statistical Analysis

Statistical analyses were performed using GraphPad Prism version 5.0 (Graph Pad Software, San Diego, CA, United States). All data are presented as the mean ± SEM and were compared using one-way ANOVA followed by the Newman-Keuls test as a post hoc test. *p* < 0.05 was considered a statistically significant difference.

## Results

### Identification of Primary Cortical Neurons From Fetal Rats

To identify neurons, cells that had been cultured for 7 days were immunostained with the neuronal marker NeuN (green), and the nuclei were stained with DAPI (blue). Six fields were randomly selected, and 90.9% ± 0.9% of cells were determined to be positive for immunofluorescent staining with both NeuN and DAPI under these conditions (data not show).

### ICT Increases Cell Viability and Reduces LDH Release Rate and Apoptosis

First, the cytotoxicity of ICT on rat cortical neurons was tested using by treating cells with different concentrations of ICT. ICT (0.1, 0.3, and 1 μM) showed no significant effect on cell viability or LDH release rate (Figures 1B,C). To study the protective effect of ICT on excitatory toxicity, a glutamate-induced neuronal injury model was generated from cortical neurons by treating them with 100 μM glutamate and 10 μM glycine for 1 h. The resulting neuronal viability was shown to be significantly decreased compared to that of the control group, as expected. These cellular damages were reverted by treatment with 0.1, 0.3, and 1 μM ICT (Figure 1B). On the other hand, glutamate treatment significantly increased the release rate of LDH compared to that of the control group. However, ICT treatment at 0.1, 0.3, and 1 μM significantly lowered the LDH release rate in cortical neurons (Figure 1C). These findings suggest that ICT acted as a neuroprotective agent in glutamate-induced neuronal injury, and a concentration of 1 μM was used for subsequent experiments. To further elucidate the anti-apoptotic capability of ICT, the percentage of apoptotic cells was detected by flow cytometry following annexin V/PI double staining. The results collected from flow cytometry are shown on scatter plots (Figure 1D). The upper left indicates annexin V^–^/PI^+^ cells (necrotic); the upper right indicates annexin V^+^/PI^+^ cells (late-phase apoptotic); the lower left indicates annexin V^–^/PI^–^ cells (viable); and the lower right indicates annexin V^+^/PI^–^ cells (early phase apoptotic). ICT treatment had no effect on the percentage of viable and apoptotic cells compared to that of the control group, which was consistent with previous results. Compared with the control group, after glutamate-induced neuronal injury, significantly fewer viable cells and a higher number of apoptotic cells were observed. With ICT treatment, the percentage of viable cells increased significantly, and fewer apoptotic cells were observed in comparison to the glutamate-only group (Figures 1D,E,F). This data reveals that ICT is a remarkable anti-apoptotic drug for glutamate-induced neuronal injury.

### ICT Reverts the Changes in Expression Levels of GluN2B and GluN2A Proteins in Glutamate-Injured Primary Cortical Neurons

**FIGURE 2:**
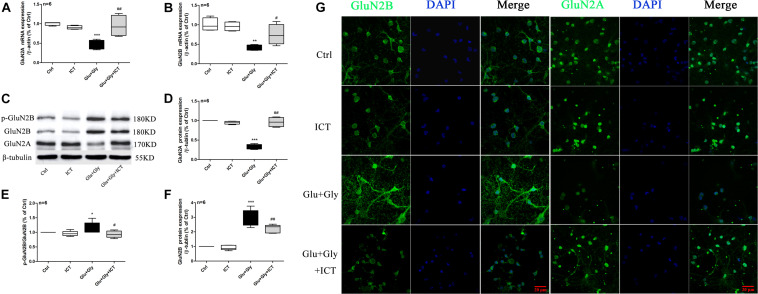
The expression levels of GluN2A and GluN2B and the phosphorylation level of GluN2B are regulated by ICT treatment after glutamate-induced injury. GluN2B and GluN2A mRNA and protein levels **(A–F)** in the different groups (control, 1 μM ICT, Glu+Gly and Glu+Gly+1 μM ICT) were analyzed by RT-qPCR and Western blot, respectively, and are expressed as the mean ± SEM (*n* = 6). GluN2B and GluN2A protein localization is shown in the immunofluorescent images **(G)**. One-way ANOVA followed by Newman-Keuls test, **(A)**: F = 10.80, **(B)**: F = 10.28, **(D)**: F = 79.78, **(E)**: F = 3.720, **(F)**: F = 55.75; *p < 0.05, **p < 0.01, and ***p < 0.001 vs. control; ^#^*p* < 0.05 and ^##^*p* < 0.01 vs. Glu+Gly.

To investigate whether ICT protects cortical neurons from excitatory toxicity injury by regulating specific subtypes of NMDAR, the expression levels of GluN2B and GluN2A in cortical neurons were detected by RT-qPCR and Western blot. The results displayed a significant decrease in the mRNA levels of both GluN2B and GluN2A after glutamate injury compared with the control group. After ICT treatment, however, the mRNA levels of these two subunits were significantly increased (Figures 2A,B). The p-GluN2B and t-GluN2B protein levels in the glutamate injury group were both significantly higher than those of the control group, and they were reduced after ICT treatment (Figures 2C,E,F). Compared to the control group, glutamate injury showed a decrease in the expression of GluN2A protein, which was returned to the normal level by ICT treatment (Figures 2C,D). Moreover, the expression of NMDAR subtypes on primary cortical neurons was detected using an immunofluorescence assay and was photographed under a confocal microscope. The images showed that higher expression levels of GluN2B and lower expression levels of GluN2A were observed on the cell membranes of the glutamate-injured cells. While ICT alone had no effect on the expression levels of GluN2B and GluN2A in the control group, it was able to revert the effect of glutamate-induced neuronal injury shown by decreased GluN2B and increased GluN2A fluorescent intensities (Figure 2G).

### ICT Returns the Expression of Proteins in the ERK/DAPK1 Pathway to Normal Levels After Glutamate-Induced Neuronal Injury

**FIGURE 3:**
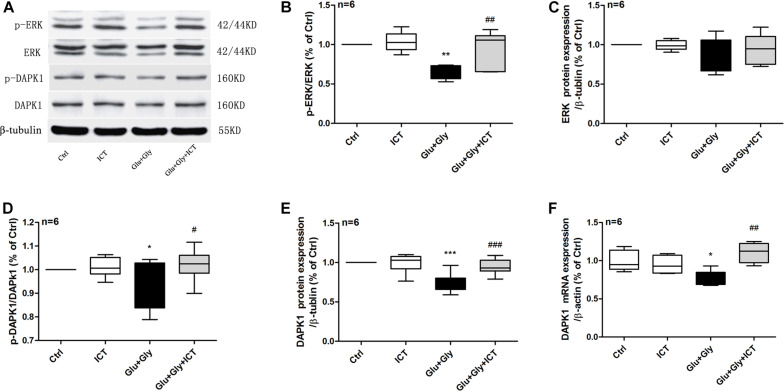
ICT rescues the reduction in phosphorylation of ERK and DAPK1 and DAPK1 expression levels after glutamate-induced neuronal injury. Western blot and RT-qPCR were used to analyze p-ERK, ERK, p-DAPK1 and DAPK1 protein expression levels **(A–E)** and DAPK1 mRNA levels **(F)** in the four treatment groups: control, 1 μM ICT, Glu+Gly and Glu+Gly+1 μM ICT, and the results are expressed as the mean ± SEM (*n* = 6). One-way ANOVA followed by Newman-Keuls test, **(B)**: F = 8.998, **(C)**: F = 1.295, **(D)**: F = 3.283, **(E)**: F = 13.22, **(F)**: F = 8.403; *p < 0.05, **p < 0.01, and ***p < 0.001 vs. control; ^#^*p* < 0.05, ^##^*p* < 0.01, and ^###^*p* < 0.001 vs. Glu+Gly.

To examine whether ICT alters the ERK/DAPK1 pathway after glutamate-induced injury, RT-qPCR and Western blotting were used to evaluate the expression levels of ERK and DAPK1. The results showed that while the phosphorylation level of ERK decreased after glutamate injury compared to that of the control group, it was reverted back to a level similar to that of the control group following ICT treatment, which was significantly higher than that of the glutamate model group. No change was observed in the total ERK protein level in any of the four experimental groups (Figures 3A–C). Similarly, the glutamate-treated group displayed significantly lower expression levels of DAPK1 mRNA, p-DAPK1 and t-DAPK1 proteins than the control group. Compared to the model group, ICT treatment significantly increased DAPK1 mRNA, p-DAPK1 and t-DAPK1 protein levels (Figures 3A,D–F).

**FIGURE 4:**
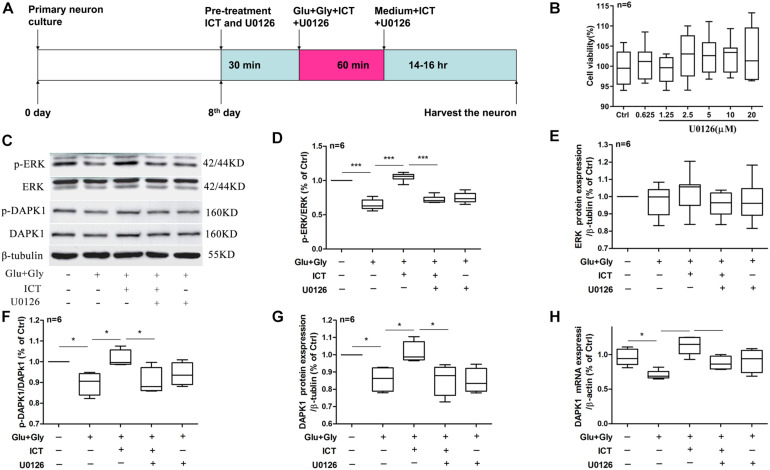
The rescuing effect of ICT on the phosphorylation of ERK and DAPK1 and DAPK1 expression post-glutamate injury is hindered by the inhibition of ERK. Timeline for the rat primary cortical neurons glutamate-induced injury model treatment with ICT and U0126 **(A)**: to clarify the role of ERK in the effect of ICT on neuroprotection and the functions or expression levels of DAPK1, GluN2B and GluN2A, primary neuronal cultures were treated with ICT and U0126, a specific inhibitor of ERK, from 30 min before glutamate injury until the cells were harvested 14–16 h after injury. The effects of different concentrations of U0126 on cell activity were detected, and the results showed that U0126 had no significant effect on the viability of normally cultured neurons **(B)**. Western blot and RT-qPCR were performed to analyze p-ERK, ERK, p-DAPK1 and DAPK1 protein expression **(C–G)** and DAPK1 mRNA levels **(H)** from five treatment groups: control, Glu+Gly, Glu+Gly+1 μM ICT, Glu+Gly+1 μM ICT+5 μM U0126 and Glu+Gly+5 μM U0126; the results are expressed as the mean ± SEM (*n* = 6). One-way ANOVA followed by Newman-Keuls test, **(D)**: F = 53.08, **(E)**: F = 0.4795, **(F)**: F = 4.966, **(G)**: F = 6.102, **(H)**: F = 8.376; *p < 0.05, **p < 0.01, and ***p < 0.001.

### The Protective Effect of ICT on Cortical Neurons Is Mediated by ERK

In order to explore the baseline effect of U0126, we designed an experiment to detect neurons viability after treatment different concentrations (0.0625, 1.25, 2.5, 5, 10, and 20 μM). It was found that U0126 (from 0.0625 to 20 μM) had no significant effect on the viability activity of normally cultured neurons (Figure 4B). Whether the neuroprotective effects of ICT on excitatory toxicity injury are mediated by the DAPK1/ERK signaling pathway was determined using Western blot and RT-qPCR experiments. The protein expression levels of p-ERK, p-DAPK1 and t-DAPK1, as well as DAPK1 mRNA, decreased significantly 14 h after excitatory toxicity injury, and ICT treatment was able to significantly recover these changes (Figures 4C,D,F–H), which was consistent with previous results. However, when the neurons were cotreated with the ERK inhibitor U0126 and ICT, the positive effects of ICT were no longer observed (Figures 4C,D,F–H). With U0126 treatment alone, there was no significant difference in p-ERK, p-DAPK1 and tDAPK1 protein levels or in DAPK1 mRNA levels when compared to those of the vehicle group (Figures 4C,D,F–H). These results suggest that ERK is the crucial player in the beneficial effect of ICT during glutamate-induced neuronal injury.

### ERK Is Required by ICT to Rescue the Expression Levels of GluN2B and GluN2A in Neurons Suffering From Excitatory Toxicity Injury

**FIGURE 5:**
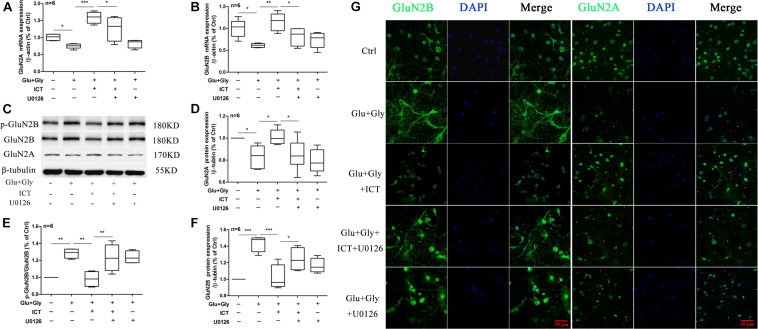
The ability of ICT to regulate GluN2B and GluN2A functions after glutamate-induced neuronal injury is diminished with the inhibition of ERK. Western blot and RT-qPCR were performed to analyze GluN2B and GluN2A mRNA and protein levels **(A–F)** from the different treatment groups: control, Glu+Gly, Glu+Gly+1 μM ICT, Glu+Gly+1 μM ICT+5 μM U0126, and Glu+Gly+5 μM U0126; the results are expressed as the mean ± SEM (*n* = 6). GluN2B and GluN2A protein localization is shown in the immunofluorescent images **(G)**. One-way ANOVA followed by Newman-Keuls test, **(A)**: F = 14.55, **(B)**: F = 6.921, **(D)**: F = 7.099, **(E)**: F = 9.517, **(F)**: F = 10.39; *p < 0.05, **p < 0.01, and ***p < 0.001.

Following excitatory toxicity injury, the expression levels of the two NMDAR subunits in different experimental groups were changed. The expression levels of both GluN2B and GluN2A mRNA were decreased significantly after glutamate injury compared with the control group, and they were significantly increased after ICT treatment, which was consistent with previous findings. However, GluN2B and GluN2A mRNA levels decreased after U0126 treatment in both the glutamate-only and glutamate with ICT treatment groups compared to groups without U0126 (Figures 5A,B). The expression of p-GluN2B and tGluN2B proteins in the glutamate group was significantly higher than that of the control group and was decreased after ICT administration. However, ICT’s effect was abolished when it was combined in treatment with U0126 (Figures 5C,E,F). The expression level of GluN2A protein was decreased significantly in the glutamate group compared with that of the control group and was rescued back to the control level after ICT administration. Similar to GluN2B, ICT’s rescuing effect on the expression level of GluN2A protein was also diminished after the administration of U0126 (Figures 5C,D). Immunocytochemistry of GluN2B and GluN2A proteins in neurons of different treatment groups provided results consistent with Western blot findings, which indicate increased expression of GluN2B and decreased expression of GluN2A in the glutamate injury model group. This observation was rescued by ICT treatment, which was reversed again by treatment with the ERK-specific inhibitor U0126 (Figure 5G). These data suggest that ERK is a crucial player in the regulatory effect of ICT on GluN2A and GluN2B expression levels post-glutamate injury.

### The Anti-apoptotic Effect of ICT on Excitatory Toxicity Injury Is Dependent on ERK Function

**FIGURE 6:**
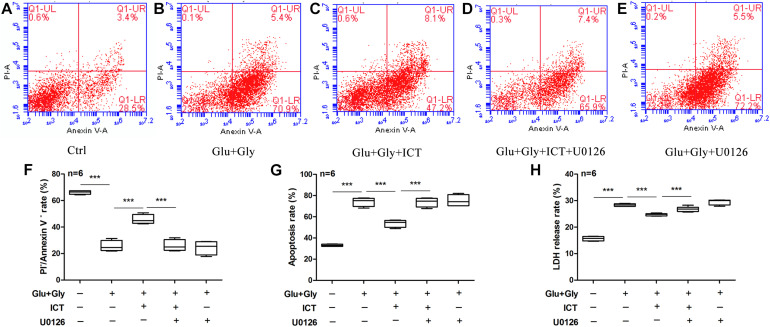
The inhibition of ERK blocks the anti-apoptotic ability of ICT in glutamate-induced neuronal injury. The annexin V/PI double staining data collected by flow cytometry are shown in scatter plots **(A–E)**. The percentage of surviving **(F)** and apoptotic **(G)** neurons and LDH release rate **(H)** from the five treatment groups (control, Glu+Gly, Glu+Gly+1 μM ICT, Glu+Gly+1 μM ICT+5 μM U0126, and Glu+Gly+5 μM U0126) are expressed as the mean ± SEM (*n* = 6). One-way ANOVA followed by Newman-Keuls test, **(F)**: F = 82.60, **(G)**: F = 82.49, **(H)**: F = 194.0; ***p < 0.001.

To further determine whether ERK is involved in the anti-apoptotic effect of ICT, apoptosis and the LDH release rate were analyzed to detect the change in cytotoxicity after glutamate injury and cotreatment with ICT and the ERK-specific inhibitor U0126. Consistent with previous findings, glutamate-induced neuronal injury significantly reduced the percentage of living cells, but the number was significantly increased after treatment with 1 μM ICT. The cotreatment of ICT and U0126 diminished the rescuing effect of ICT. In addition, the ability of 1 μM ICT to significantly reduce the percentage of apoptotic cells in the glutamate-injured model group was abolished by the administration of U0126 (Figures 6A–G). Moreover, the LDH release rate in the cortical neurons after glutamate-induced injury and ICT treatment was significantly lower than it was in the glutamate-only group, but it was increased again after U0126 treatment (Figure 6H). These findings revealed that the inhibition of ERK disrupts the neuroprotective effect of ICT in glutamate-induced neuronal injury.

## Discussion

Several studies have shown that icaritin plays a protective role in the nervous system, mainly because of the following: icaritin can inhibit the inflammatory response and oxidative stress of hippocampal neurons, which might be realized through the TLR4-NF-kappa B signaling pathway (Xu et al., 2009; Zhu et al., 2010, 2019). Icaritin could significantly reduce the learning and memory impairment in rats induced by D-gal, and its mechanism might be related to antioxidant stress and the reduction of BDNF and TrkB protein expression (Liu et al., 2019). Icaritin also protects mitochondrial function in 3x Tg-AD mice, regulates brain energy metabolism and improves cognitive function in 3x Tg-AD mice (Chen et al., 2016). As phytoestrogens, their neuroprotective effects may be related to estrogen receptors. Icaritin showed neuroprotective effects in primary rat neuronal cell cultures from amyloid beta-mediated neurotoxicity via an estrogen-dependent pathway (Wang et al., 2007). Icaritin promoted the coexpression of beta-tubulin III and choline acetyltransferase in neurons from mouse embryonic stem cells in an estrogen receptor-independent manner (Wang et al., 2009). In this study, we revealed the role of ICT in opposing excitatory toxicity and its mechanism.

Neuroexcitatory toxicity induced by excessive glutamate release has been the major focus in the study of cerebral ischemia/reperfusion injury. The NMDA glutamate receptor has been shown to be responsible for mediating excitotoxicity (Martin and Wang, 2010; Yang et al., 2010; Liu et al., 2012; Zhang et al., 2013; Fan et al., 2015; Yuan et al., 2015; Vieira et al., 2016). In this study, we show that icaritin (ICT) protects rat cortical neurons from glutamate-induced neuronal injury *in vitro*. This neuroprotective effect of ICT was found to be dependent on ERK, suggesting its possible mechanism of action.

In the current study, a glutamate-induced neuronal injury model was used to induce excitatory toxicity injury in rat primary neuronal cultures; the model was established by cotreatment with 100 μM glutamate and 10 μM glycine. This model was able to cause neuronal damage, which manifested as a decreased neuron survival rate and an increased rate of LDH release and apoptosis (Figure 1). ICT treatment significantly reduced glutamate-induced neuronal damage but showed no cytotoxicity in the absence of glutamate. These results suggest that ICT is a potential neuroprotective agent that is capable of ameliorating excitatory toxicity induced by glutamate.

Functional NMDA receptors are mostly composed of GluN1 and different GluN2 subunits. GluN2 is a regulatory subunit with glutamate binding sites, and the GluN2A and GluN2B subunit subtypes play different roles in cerebral ischemia/reperfusion injury. Activation of GluN2A-containing NMDA receptors promotes neuronal survival (Choo et al., 2012; Lai et al., 2014; Li et al., 2015; Huang et al., 2017a; Khoshnam et al., 2017; Shi et al., 2017; Sun et al., 2018a), while activation of GluN2B-containing NMDA receptors causes excitatory toxicity, and its mechanism is mainly related to apoptosis induced by Ca^2+^ overload in neurons (Wang et al., 2013). Therefore, improving GluN2A function and inhibiting GluN2B function are considered the most favorable mechanisms for alleviating ischemic injury (Shu et al., 2014). Our results showed that ICT treatment rescues the mRNA and protein expression levels of GluN2A and reduces the protein levels of p-GluN2B and t-GluN2B after glutamate-induced injury (Figure 2), suggesting that ICT inhibits neuronal damage by regulating the expression and phosphorylation profiles of the subtypes of the NMDAR subunits. However, ICT also increased the mRNA level of GluN2B, which may be due to the negative feedback regulation due to the low protein level of GluN2B. The underlying mechanism behind the ability of ICT to lower GluN2B protein levels needs to be further explored.

Death-associated protein kinase 1 (DAPK1), a key molecule in the neuronal death cascade, phosphorylates GluN2B at Ser1303 and enhances the conductance of GluN2B-containing NMDA receptors (Sun et al., 2018b), leading to irreversible neuronal death. Phosphorylated DAPK1 (p-DAPK1) is inactive, while dephosphorylated DAPK1 is active and binds to the GluN2B subunit (Liu et al., 2012). Tu et al. (2010) revealed that DAPK1 found in the cerebral cortex phosphorylates GluN2B at S1303 during cerebral ischemia (Li et al., 2017b; Wang et al., 2017). As a result, it increases the permeability of GluN2B-containing NMDA receptors, promotes Ca^2+^ influx and ultimately causes ischemic neuronal death (Martin and Wang, 2010; Tu et al., 2010; Zhang et al., 2015; Liu et al., 2016). Blocking the interaction between DAPK1 and GluN2B has been proven to inhibit neuronal death (Tang et al., 2018), and the deletion of the DAPK1 gene blocks Ca^2+^ influx and protects neurons from cerebral ischemic injury (Tu et al., 2010). Thus, increasing the level of DAPK1 phosphorylation inhibits the activation of GluN2B-containing NMDARs and improves neuronal outcomes after glutamate-induced injury.

**FIGURE 7:**
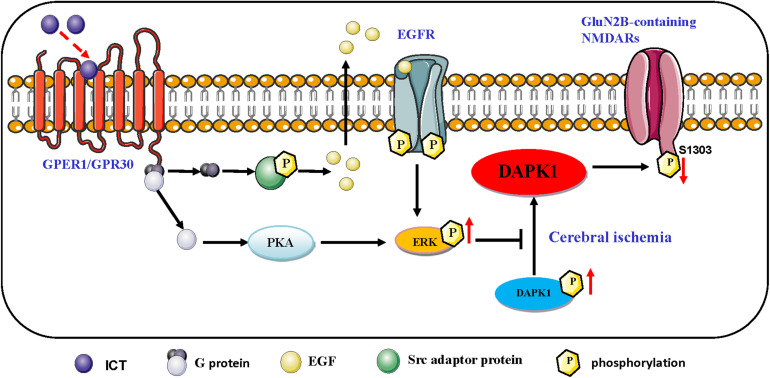
Schematic representation of the proposed resisting the excitability toxicity signaling pathways triggered by ICT in ischemic neurons. ICT activates GPR30-ERK pathways, and phosphorylated ERK up-regulate the phosphorylated DAPK1, which prevent the phosphorylation of GluN2B and the activation of GluN2B-containing NMDAR.

Extracellular signal-regulated kinase 1/2 (ERK1/2) is a member of the mitogen-activated protein kinase (MAPK) family, and it is the most common activator of MAPK. Studies have shown that after activation of the protein by phosphorylation, ERKs translocate into the nucleus and modulates cell function, including promoting cell proliferation and survival (Li et al., 2016; Xiong et al., 2018). The ERK1/2 signaling cascade is a crucial pathway that mediates NMDA receptor plasticity and maintenance (Liu et al., 2017). Activation of the ERK1/2 cascade has a neuroprotective role, as it is essential to the normal expression of NMDA receptors as well as for inhibition of GluN2B phosphorylation and reduction of the abnormal functioning of NMDA receptors. Liu and colleagues (Liu and Zhao, 2013) proposed that the ERK1/2 signaling pathway reduces the phosphorylation of DAPK1, thereby blocking GluN2B dephosphorylation, downregulating the activation of NMDA receptors and obtaining cerebral protection effects. Our study found that ICT increases the phosphorylation levels of ERK1/2, DAPK1 and GluN2A and reduces the phosphorylation of GluN2B (Figures 2, 3); collectively, these may be the mediators of the protective effect of ICT against glutamate-induced neuronal damage. When the ERK-specific inhibitor U0126 was applied to neurons at the same time as ICT, the effects of ICT on the ERK/DAPK1 pathway and NMDAR subunits were abolished (Figures 4, 5) and so were its ability to reduce the rate of apoptosis and cytotoxicity (Figure 6). This indicates that ERK is a crucial mediator in the mechanism of ICT action. The above results suggest that ICT may prevent the phosphorylation of GluN2B and enhance the expression of GluN2A by activating ERK1/2/DAPK1 signaling, therefore inhibiting excitatory toxic injury induced by glutamate and protecting neuronal cells against cell death (Figure 7).

The key pathway of synaptic plasticity is the activation of n-methyl-D-aspartate receptor (NMDAR) signals connected to the ERKs cascade. ERK dependence has been shown to exist in many forms of synaptic plasticity and in learning and memory, including local synaptic processes and long-range signals to the nucleus (Melgarejo et al., 2016). However, it is not clear whether ERK is upstream or downstream. In this study, icaritin eventually played a neuroprotective role by regulating the expression and/or activation of DAPK1, GluN2B and GluN2A. Some studies indicated that GluN2B/CaMKII could regulate the activation of ERK in the upstream of ERK (Cao et al., 2012; El Gaamouch et al., 2012; Melgarejo et al., 2016; Qiu et al., 2017; Buonarati et al., 2020). DAPK1 could bind to GluN2B regions, which required allosteric regulation of CaMKII (Cao et al., 2012; Goodell et al., 2017; Tu et al., 2018; Buonarati et al., 2020). We proposed the hypothesis that the relationship between ERK and GluN2B may be a bidirectional regulatory relationship, in which DAPK1/GluN2B can regulate the activation of ERK, while ERK can regulate the phosphorylation of DAPK1 and the expression of GluN2B. At present, there is no relevant report about the possible bidirectional regulation effect of ERK and GluN2B, and this is the first discovery. How activated ERK regulates the expression of DAPK1 and MDARs is not yet clear.

Icaritin is a phytoestrogen with estrogen-like active structure and induces its effects through activation of estrogen membrane receptors and nuclear receptors (Wu et al., 2017). G-protein-coupled estrogen receptor (GPER) is widely distributed in various tissues of the body. In the nervous system, GPER is mainly distributed in the cortex of the forebrain, hypothalamus, hippocampus, the locus coeruleus of the midbrain, trigeminal nucleus, and cerebellar Purkinje cells, which is involved in learning, memory, and cognition (Liu and Zhao, 2013). Activating GPER activates ERK1/2 signaling through PKA or Epidermal growth factor (EGF)/ Epidermal growth receptor (EGFR) (Zhang et al., 2016; Figure 7).

GPER, together with intracellular estrogen receptor, activates cell signal transduction, which is considered as one of the important pathways to promote the survival of neurons after cerebral ischemia. Estrogen or GPER agonist G1 can inhibit excitatory toxic injury induced by NMDA exposure, which is achieved by inhibiting dephosphorylation of p-DAPK1, thereby inhibiting phosphorylation of GluN2B (Liu et al., 2012). Combined with the above evidence, Icaritin may up-regulation of ERK signaling through GPER, which needs to be confirmed by further experiments.

## Conclusion

In conclusion, ICT offers a therapeutic advantage of protection against glutamate-induced neuronal damage. Notably, changes in the functions of GluN2A-containing and GluN2B-containing NMDARs mediated by the ERK/DAPK1 pathway may be responsible for the effects of ICT on excitatory toxic injury. These data provide strong evidence for the neuroprotective potential and a mechanistic basis for ICT targeting cerebral ischemic injury and ischemic encephalopathy. This study provides new clues for the prevention and treatment of clinical ischemic cerebrovascular disease and encourages further investigations to evaluate the therapeutic potential of ICT in neurological diseases.

## Data Availability Statement

All datasets generated for this study are included in the article/supplementary material.

## Ethics Statement

The animal study was reviewed and approved by Ethics Committee of Biomedical Research of Gannan Medical University.

## Author Contributions

SL designed the experiments. SL works in Xiamen Haicang Biological Science and Technology Development after graduating from GMU. SL, CL, JX, and LX carried out the experiments. CH analyzed experimental results. RP, ZH, and LL wrote the manuscript. All authors contributed to the article and approved the submitted version.

## Conflict of Interest

The authors declare that the research was conducted in the absence of any commercial or financial relationships that could be construed as a potential conflict of interest.
